# On the durability of surgical masks after simulated handling and wear

**DOI:** 10.1038/s41598-022-09068-1

**Published:** 2022-03-23

**Authors:** Vincent Varanges, Baris Caglar, Yann Lebaupin, Till Batt, Weidong He, Jing Wang, René M. Rossi, Gilles Richner, Jean-Romain Delaloye, Véronique Michaud

**Affiliations:** 1 Laboratory for Processing of Advanced Composites (LPAC), Institute of Materials (IMX), Ecole Polytechnique Fédérale de Lausanne (EPFL), Station 12, 1015 Lausanne, Switzerland; 2 Aerospace Manufacturing Technologies, Faculty of Aerospace Engineering, Delft University of Technology, Kluyverweg 1, Delft, 2629HS The Netherlands; 3 Biomimetic Membranes and Textiles Laboratory, Swiss Federal Laboratories for Materials Science and Technology (Empa), 9014 St. Gallen, Switzerland; 4 Laboratory of Advanced Analytical Technologies, Swiss Federal Laboratories for Materials Science and Technology (Empa), 8600 Dübendorf, Switzerland; 5 Institute of Environmental Engineering, ETH Zürich, 8093 Zürich, Switzerland; 6 Spiez Laboratory, Federal Office for Civil Protection FOCP, Spiez, Switzerland; 7 Department of Surgery, Clinic of Orthopaedics and Traumatology, Kantonsspital, Winterthur, Switzerland

**Keywords:** Viral infection, Polymers, Mechanical engineering

## Abstract

After the spread of COVID-19, surgical masks became highly recommended to the public. They tend to be handled and used multiple times, which may impact their performance. To evaluate this risk, surgical masks of Type IIR were submitted to four simulated treatments: folding, ageing with artificial saliva or sweat and washing cycles. The air permeability, mechanical integrity, electrostatic potential, and filtration efficiency (FE) of the masks were measured to quantify possible degradation. Overall, air permeability and mechanical integrity were not affected, except after washing, which slightly degraded the filtering layers. Electrostatic potential and FE showed a strong correlation, highlighting the role of electrostatic charges on small particle filtration. A slight decrease in FE for 100 nm particles was found, from 74.4% for the reference masks to 70.6% for the mask treated in saliva for 8 h. A strong effect was observed for washed masks, resulting in FE of 46.9% (± 9.5%), comparable to that of a control group with no electrostatic charges. A dry store and reuse strategy could thus be envisaged for the public if safety in terms of viral and bacterial charge is ensured, whereas washing strongly impacts FE and is not recommended.

## Introduction

The novel coronavirus disease (COVID-19) has recently spread worldwide. The virus, 65–125 nm^1^ in diameter, infects the respiratory tract and leads to symptoms such as cough, fever, dyspnea and tiredness^2^. Spreading of the disease is therefore eased from human to human through respiratory droplets and aerosols^3^ that are produced, among other ways, by coughing or sneezing. In order to hinder airborne virus propagation, surgical mask-wearing has become highly recommended to the population, rapidly leading to a global mask shortage^4^ during the first months of the pandemic. Alternative solutions such as textile masks, including homemade and improvised masks, were quickly developed, but these tend to show a reduced filtration efficiency^5,6^. The shortage of surgical masks partly resulted from their initial purpose reserved for hospital use, recommending not to wear the mask for more than 4 h and to dispose it after use^7^. With the increasing adoption of surgical masks by the public, who is generally less directly exposed to the virus, the question of the durability of masks in terms of mechanical resistance and filtration efficiency needs to be further evaluated. Indeed, many users tend to wear masks intermittently during the day, to fold and store them in various forms, whereas some have even started to wash them for further reuse, following recommendations in the press and the urge to limit plastic waste.

The filtration of airborne droplets relies on gravity sedimentation, inertial impaction, interception, diffusion and electrostatic attraction^8^. These combined mechanisms make it possible for the multi-layered non-woven polymer structure to reach, for pristine masks, a filtration efficiency of 99.5% for particles larger than 300 nm and 75% for particles smaller than 300 nm^9^. During use, handling and potentially washing, surgical masks may be subject to loss of mechanical integrity or to electrostatic charge decay that would reduce their filtration performance^10^. The effect of washing has been recently evaluated by a consortium of researchers in France, by quantifying the change in bacterial filtration efficiency (BFE), which correspond to a particle size of 3 ± 0.3 µm, as recommended by the EN 14683 norm for surgical masks. They concluded that washing had an effect on electrostatic interactions, but had no quantifiable effect on BFE and breathability^11^. They however did not focus on the effect of mechanical handling and folding, as well as contact with body fluids on the performance of the masks.

**Figure 1:**
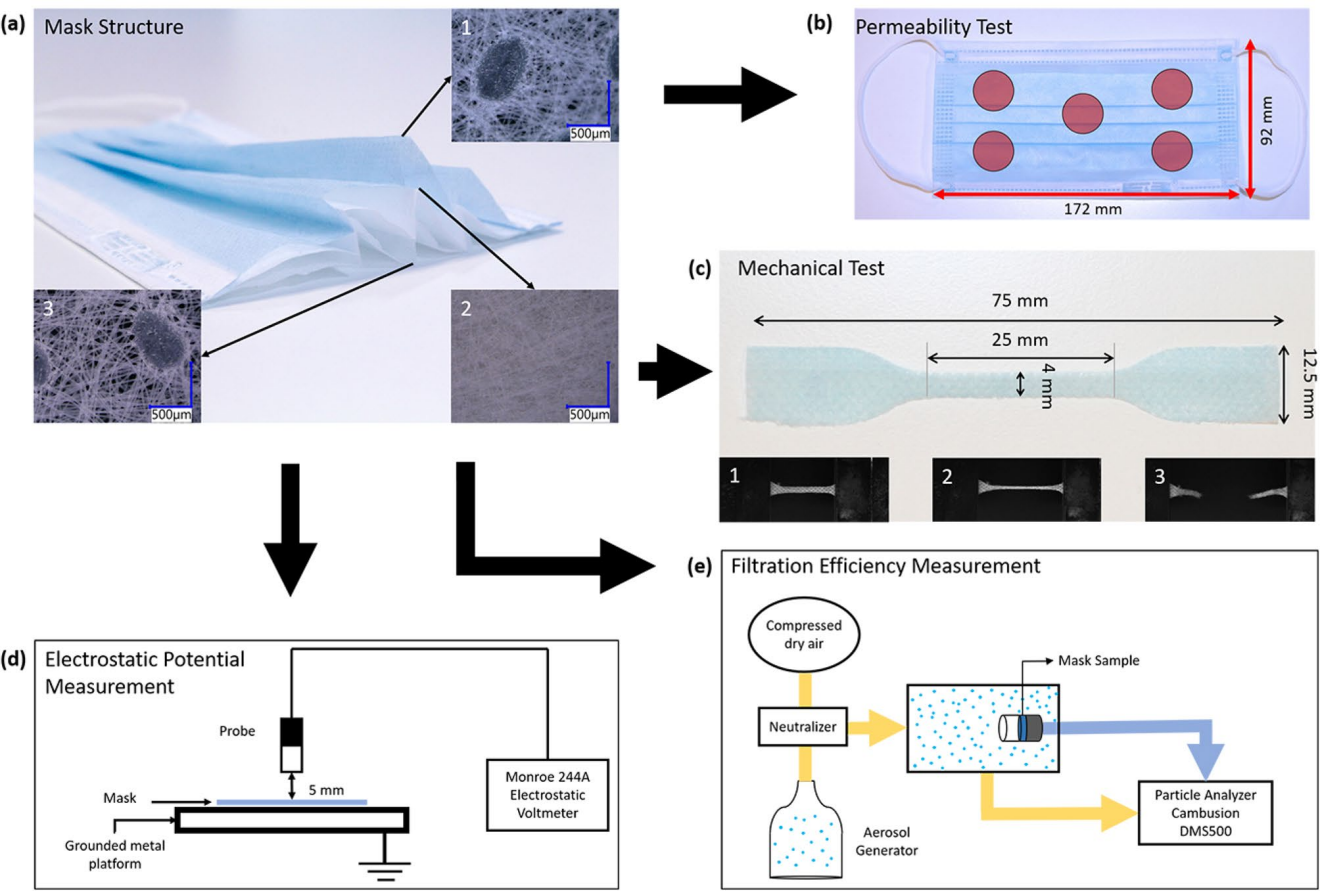
Schematics of mask durability characterization methods: (**a**) three layer configuration of a Type IIR surgical mask along with the micrographs of the (1) outer layer, (2) the middle layer and (3) the inner layer; (**b**) locations of the permeability measurements performed on each mask; (**c**) dimensions of the samples for the mechanical (tensile) test punched out from the masks along with pictures (1) before, (2) during and (3) after the test; (**d**) mask electrostatic potential measurement method and (**e**) filtration efficiency measurement method.

We thus propose to quantify the influence of handling and wearing on mask integrity and filtration efficiency, using laboratory-scale protocols which mimic extreme scenarios of masks’ manipulation and wear, including repeated folding experiments, storage in artificial sweat and saliva and finally washing events. These worst-case scenarios are summarized in Table 1 and Fig. 1 provides micrographs of the mask structure with three layers and a visual summary of the performed tests.

**Table 1:** List of all the configurations performed during this study classified by type of treatment with the number of masks treated.

Folding	Ageing		Ageing + Folding		Combined in-situ		Isopropanol
	Saliva	Sweat	Saliva	Sweat	Saliva	Sweat	
10 Cycles (1 mask)	1 Hour (1 mask)		1 Hour + 10 Cycles (1 mask)		10 Cycles (1 mask)		1 h (1 mask)
20 Cycles (1 mask)	4 Hours (1 mask)		4 Hours + 20 Cycles (1 mask)		20 Cycles (1 mask)		
50 Cycles (1 mask)	8 Hours (1 mask)		8 Hours + 50 Cycles (1 mask)		50 Cycles (1 mask)		
100 Cycles (3 masks)	1 week (3 masks)

## Results

### Air permeability

**Figure 2:**
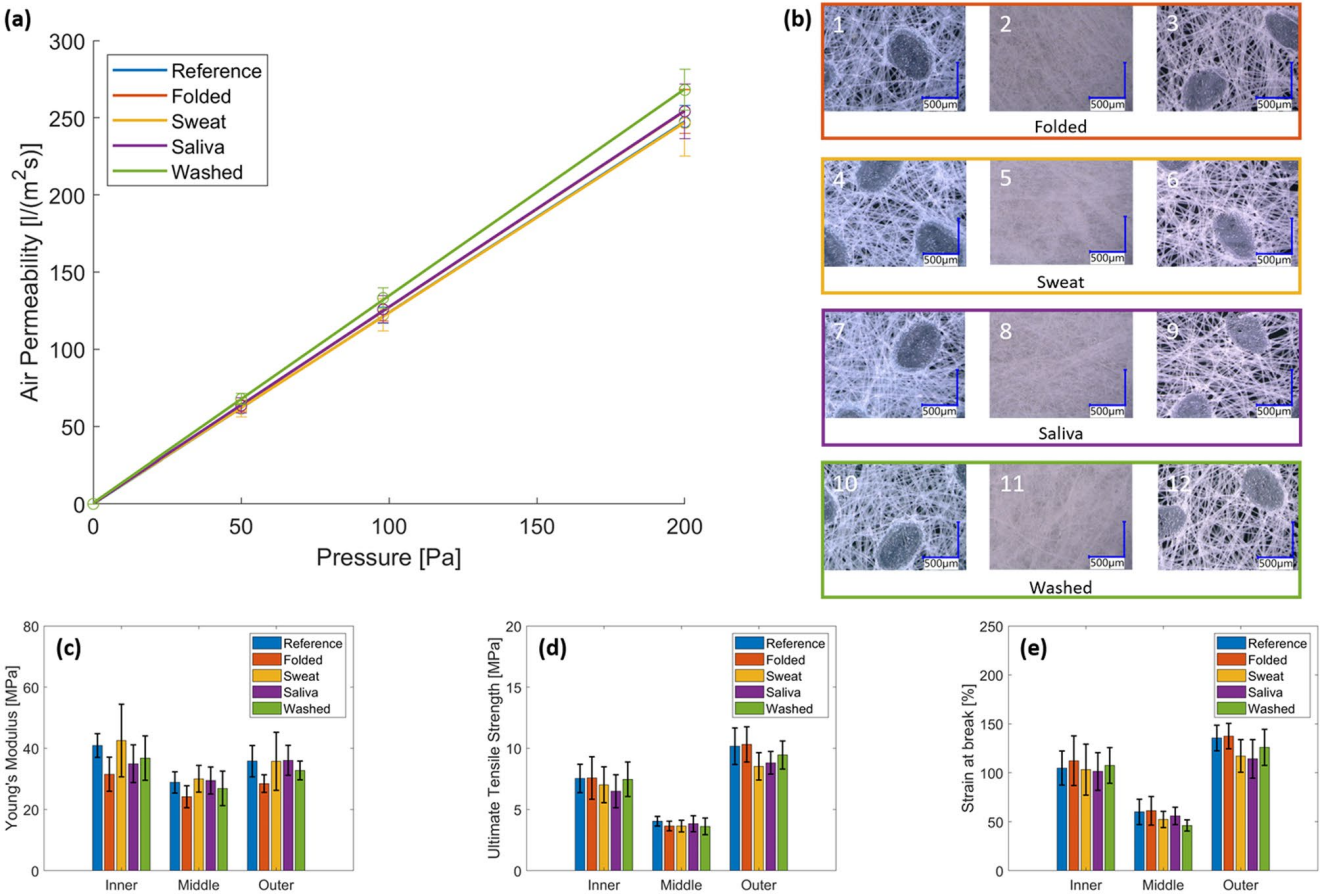
Characterization of the mask integrity after strong treatments: (**a**) Fitted curves of air permeability as a function of input pressure for reference and treated masks; (**b**) Micrographs of the outer (1) (4) (7) (10), middle (2) (5) (8) (11) and inner (3) (6) (9) (12) layers for the four treatment types; from mechanical testing: Young’s modulus (**c**), the ultimate tensile strength (**d**) and the strain at break (**e**) were derived for each layer of the reference (untreated) and treated masks.

Figure 2a displays the permeability values as a function of input pressure for masks with the various treatments. The figure shows little scatter for all five different mask types and a linear dependency on the test pressure, with a correlation coefficient of 0.98 and 1. The air permeability values lie in the same range for all the treatments, except for the washed masks which show a slightly higher permeability overall. Data thus indicate that the severe folding and ageing treatments endured by the masks do not modify the air permeability of the fabric.

**Figure 3:**
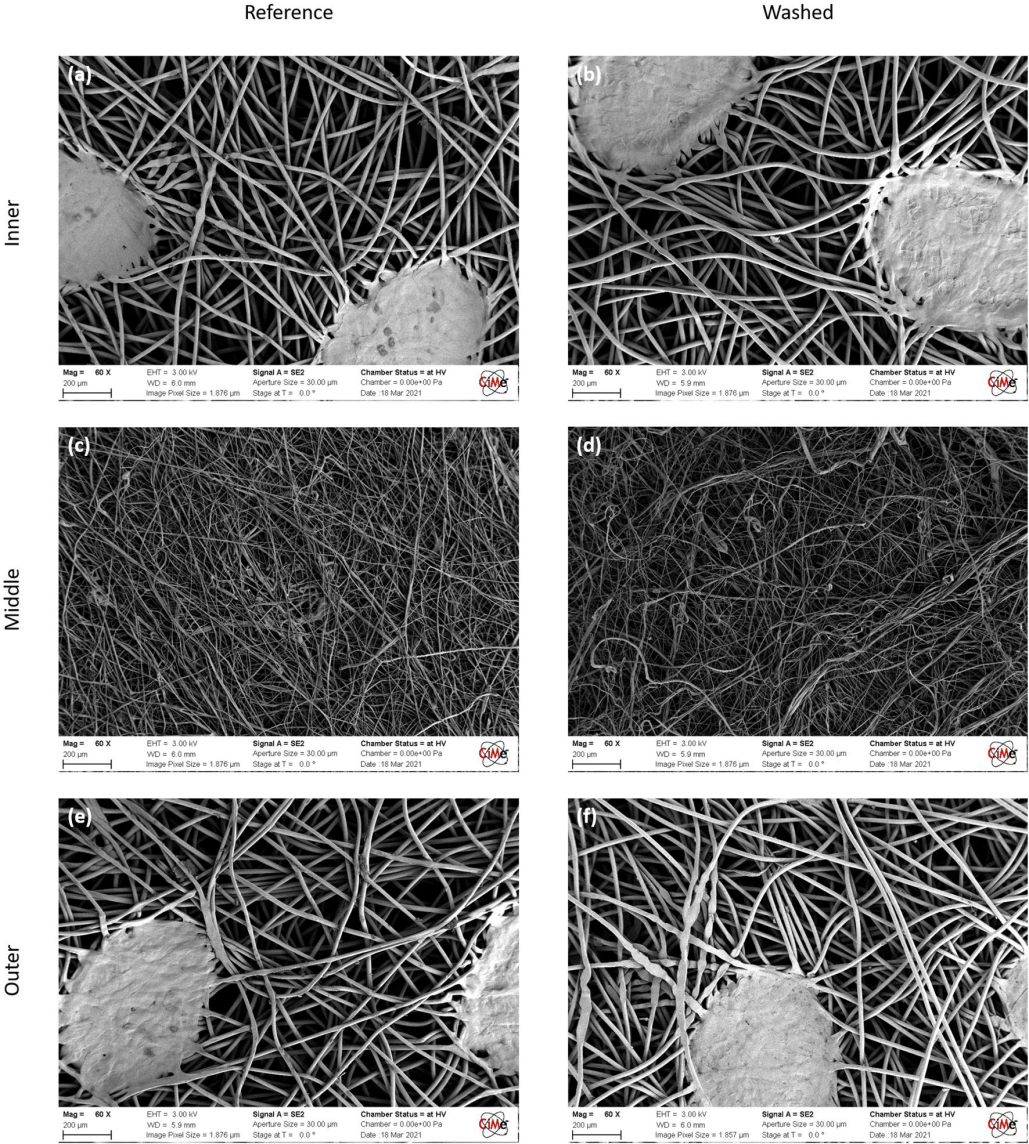
SEM micrographs of each layer from a reference and washed mask: (**a**, **b**) inner layer made of spunbond PP fibers with diameters of 15–20 microns, (**c**, **d**) middle layer made of melblown fibers with diameters of 1–3 microns, (**e**, **f**) outer layer made of spunbond PP fibers as the inner layer.

Table 2 reports the pressure drop induced by the masks according to EN14683. For all configurations, the average ΔP was between 41.4 and 46 Pa cm^−2^, below the 60 Pa cm^−2^ stated in EN14683 with the lowest value in the case of the washed masks. Figure 2b displays the micrographs obtained after each treatment, whereas Fig. 3 displays SEM micrographs of a reference and washed mask. A clear impact of washing is observed on the middle layer whereas the inner and outer layers remain identical before and after washing.

**Table 2:** For each type of treatment, list of the coefficient of determination R^2^ of the permeability fitted curves in Fig. 2a along with the derived Pressure drop for an airflow of 8 L min^−1^. In the last three columns, details of the derived mechanical properties for each treatment and layer: Young’s modulus, ultimate tensile strength and strain at break (also presented in Fig. 2c–e).

Treatment	Layer	Air permeability coefficient of determination R^2^ [–]	Pressure Drop [Pa cm^−2^]	Young’s modulus [MPa]	Ultimate tensile strength [MPa]	Strain at break [%]
Reference	Inner	0.996	44.9 ± 1.9	40.9 ± 3.9	7.5 ± 1.2	104.8 ± 17.4
	Middle			28.8 ± 3.5	4.0 ± 0.4	60.0 ± 13.0
	Outer			35.8 ± 5.1	10.2 ± 1.5	135.5 ± 13.2
Folded	Inner	0.993	43.8 ± 2.5	31.5 ± 5.6	7.6 ± 1.7	112.2 ± 25.3
	Middle			24.2 ± 3.6	3.7 ± 0.4	61.2 ± 14.6
	Outer			28.5 ± 2.9	10.3 ± 1.4	137.4 ± 13.0
Sweat	Inner	0.984	45.3 ± 4.0	42.6 ± 11.9	7.0 ± 1.5	103.3 ± 26.1
	Middle			30.0 ± 4.4	3.6 ± 0.5	52.3 ± 8.2
	Outer			35.7 ± 9.5	8.5 ± 1.1	117.2 ± 16.6
Saliva	Inner	0.989	43.8 ± 3.0	35.0 ± 6.2	6.5 ± 1.3	101.3 ± 19.2
	Middle			29.4 ± 4.4	3.8 ± 0.7	55.9 ± 8.8
	Outer			36.1 ± 4.9	8.8 ± 0.9	114.2 ± 19.7
Washed	Inner	0.994	41.4 ± 2.1	36.8 ± 7.3	7.5 ± 1.4	107.6 ± 18.2
	Middle			26.9 ± 5.6	3.6 ± 0.7	46.2 ± 5.8
	Outer			32.8 ± 3.1	9.5 ± 1.2	126.0 ± 18.4

### Mechanical performance

Figure 2c–e report the mechanical properties as a function of the layer and treatment. Young's moduli (as defined in the “Methods” section) of inner and outer layers (40.9 MPa and 35.8 MPa) are slightly higher than that of the middle layer (28.8 MPa), in the range reported by Ekabutr et al.^12^. This pattern repeats itself for the treated masks as observed in Fig. 2c and in Table 2. The middle layer of the washed masks shows a slightly lower strain at break when compared to the other treatments (Fig. 2e), whereas folding and ageing treatments do not significantly alter the mechanical properties of the three layers forming the mask structure.

### Electrostatic potential measurements

**Figure 4:**
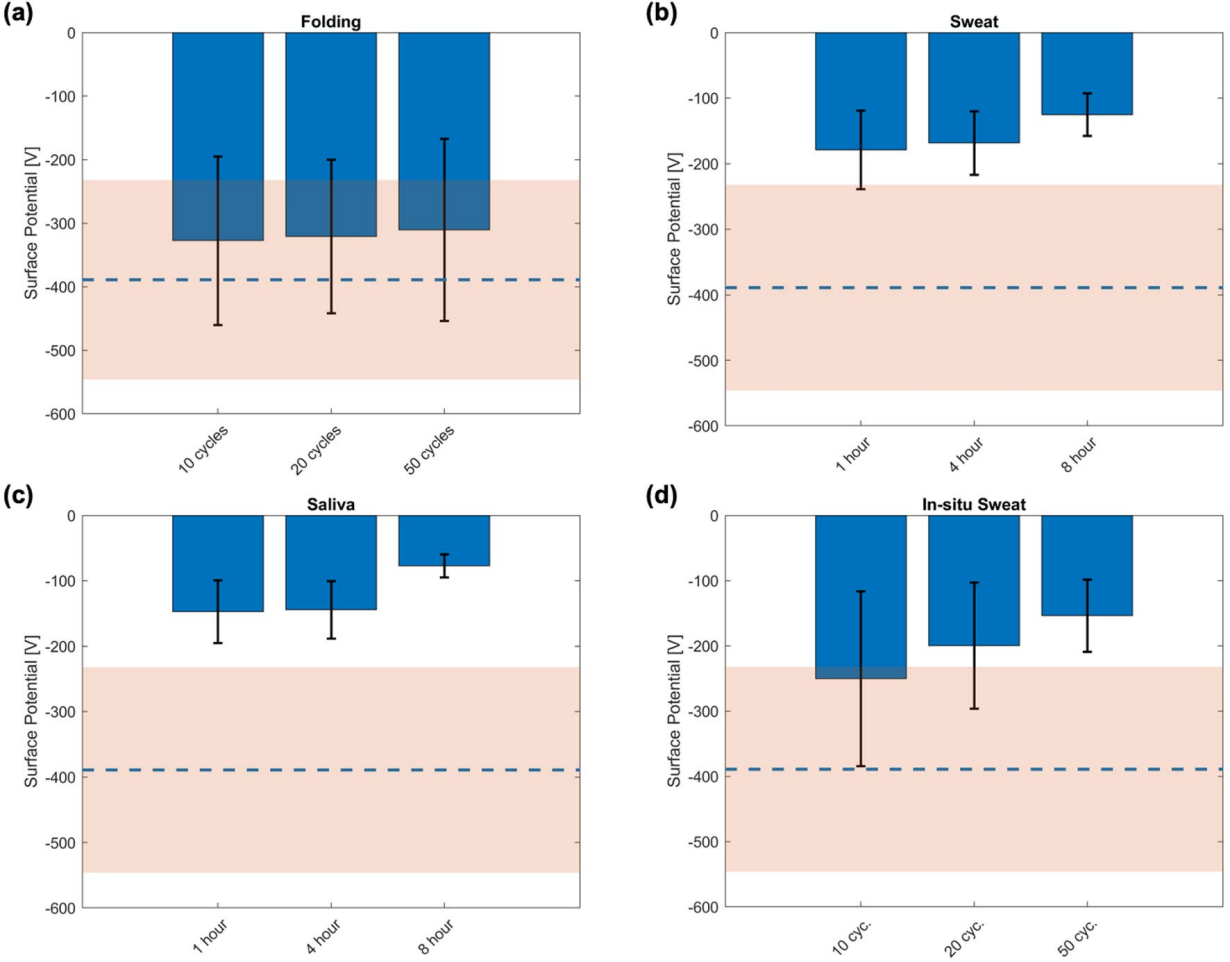
Characterization of the surgical mask’s surface potential (electrostatic potential): For each figure the reference data are used as a comparison and are represented by the dashed blue line with its standard deviation in light orange shaded area: (**a**) the folded masks; (**b**) the masks aged in sweat; (**c**) the masks aged in saliva, (**d**) the in-situ treatment with sweat.

Surface potential results are displayed in Fig. 4 and Supplementary Fig. S1. High variability is observed in most of the studied cases, attributed to the measurement technique and multiple sample handling, from the manufacturing, boxing, shipping and unboxing^13^. The reference masks (Supplementary Fig. S1a) themselves also display a strong standard deviation. The mean value varies between − 484 and − 375 V with a global standard deviation of 127 V. These values were used as the point of comparison for the rest of the measurements, represented by a dashed blue line for the mean and a light orange shaded area for the standard deviation in the figures. The results of the different folding treatments indicate a slight shift in the level of the electrostatic potential (Fig. 4a), which does not increase with the number of folding cycles. The saliva and sweat treatments had a greater impact on the electrostatic charge of the masks; the effect increases with the duration of immersion, as shown in Fig. 4b, c. The combination of two treatments (i.e., saliva or sweat plus folding) has a similar impact as sweat and saliva treatments only. On the other hand, in-situ treatments have a considerable effect on the electrostatic potential of the masks considering that they were not directly immersed in the solution, but only sprayed (Fig. 4d; Supplementary Fig. S1d). The electrostatic potential of the washed samples was not measured as the treatment was expected to remove all the charges from the masks^10^.

### Filtration Efficiency (FE)

**Figure 5:**
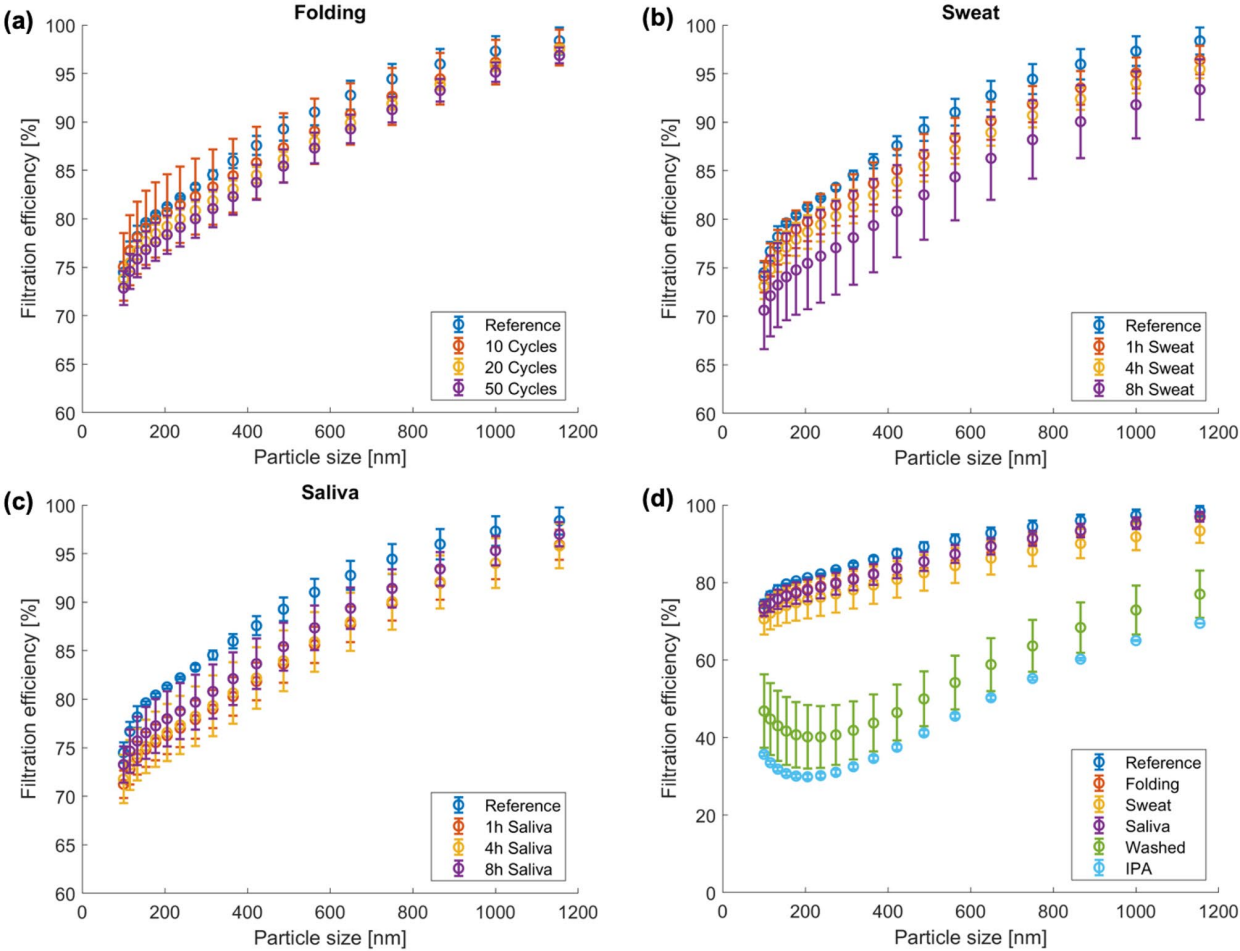
Characterization of the surgical mask’s filtration efficiency after a set of different treatments in order to assess their degree of degradation and so their durability after a given time of use: For each figure the reference data are used as a comparison and are represented by the series of blue points: (**a**) the folded masks; (**b**) the masks aged in sweat; (**c**) the masks aged in saliva; (**d**) comparison of the filtration efficiency at a different scale of the surgical masks type IIR after the different treatment, adding the FE after the washing cycles and the discharging procedure with IPA.

Figures 5 and S2 display FE results for each treatment. Figure S2a shows the FE of the reference masks together with the deviation within the same batch. Mask FE follows a similar trend as the surface potential modification after the various treatments. The folding cycles have a small effect on the FE (Fig. 5a) whereas sweat and saliva ageing induce a decrease of FE for sub-micron particle sizes (Fig. 5b, c). The same effect is noticed on the FE analyses performed after in-situ treatments, with a decrease from 97 to 92% and 93% at 1 µm for sweat and saliva, respectively. The results of the washing cycles on Fig. 5d show a strong effect on the global FE of the mask: a large drop comparable to that induced by the discharge storage in liquid isopropanol is noticed with a global loss of 35% in filtration efficiency.

## Discussion

Based on the breathability tests, microstructural observation, and mechanical test results (Fig. 2), we show that folding and body fluid treatments do not significantly impact the microstructure nor the mechanical integrity of the mask. All results on the treated masks, however, show an increased variability as compared to reference data; this is attributed to handling and manipulation of the masks. The micrographs confirm these results, showing a similar fiber distribution and pore size. The mechanical integrity of the whole mask is provided by the outer and inner layers as the middle layer can sustain significantly lower levels of stress. While most treatments do not yield any statistically significant change, the 100 folding cycles led to a degradation of Young’s modulus by 25% with respect to the reference values, indicating a loss in connectivity of the fiber network. The washed masks exhibit a slight increase in air permeability (8.5% at 200 Pa), implying a change in their integrity. The micrographs of the middle layer of the washed mask in Fig. 3, show a non-uniform fiber distribution and a larger pore size compared to the reference; likely this is the cause for the observed increase in air permeability and lower strain at break of the layer.

As the electrostatic potential is one of the main filtering mechanisms for particles of diameter below 300 nm^14^, the filtration performance of the masks and the measured electrostatic potential are strongly correlated. The folding treatment has no effect on the mask’s mechanical integrity and no charge exchange could have occurred, so the FE is not impacted. Adversely, sweat and saliva impacted both the electrostatic potential and the FE after 8 h of ageing. Neither of the two solutions is pH neutral; the saliva is acidic with a pH of 6 while the sweat is more basic within the range of 8, implying that respectively excess H^+^ and OH^-^ ions are present in these solutions. Biermann et al.^15^ suggested that charge exchange between the charges of the mask and the ions of the solutions could take place; this could also be due to an increase in the charge mobility caused by a higher electrical conductivity of the solution^16^. The in-situ treatment has a considerable effect on the electrostatic potential and the FE (Figs. 4, 5). This could be induced by the spraying while exposing the pores during folding, and by the liquid being pushed into the mask during the compression phase, inducing a full coating of the mask fibers by the solution/body fluid.

The washed masks and the isopropanol treated ones are comparable in their strong loss in FE. Isopropanol is known to suppress all the electrostatic charges; by comparison with the FE of the washed masks, it is confirmed that the charges are also annihilated in this scenario^10^. Washed mask fabrics become inefficient for the filtration of fine aerosols. Washing is thus not recommended, due to the loss in fine particles filtration, even if the filtration of larger particles, above 3 µm as recommended in the BFE measurement is not affected^11^, whereas prolonged wear and handling can be accepted if the risk of viral contamination is not high.

This study provides an evaluation of the durability of Type IIR medical masks under prolonged public use, above the usual recommendation of 4-h wear time and without an immediate discarding the mask. The methods developed for this study are based on our interpretation of potential mask usage adapted to reproducible laboratory conditions. To ensure comparison of the masks’ filtration efficiency results for each treatment, all masks were taken from the same batch and same provider as this measurement is highly dependent on the mask source and lot number. Therefore, this study only highlights the relative effects of handling and wear on the efficiency of the mask, for a commercial mask of good initial intrinsic quality. To keep the limit of one batch testing, only one mask was tested per scenario for the filtration efficiency and three samples were cut from each mask, restricting the statistical value of the study, which was meant to provide first quantifications and evaluation of risks. Finally, this study only assessed the role of the polypropylene (PP) non-woven material and did not consider mask fitting related issues, or any viral contamination effects.

As a conclusion, we assessed the durability of Type IIR surgical masks after simulated use, probing the possibility of prolonged use in case of mask shortage, as well as limiting the environmental burden of this single-use plastic by increasing its use time^17,18^. The main outcomes of the current study are thus that ageing in saliva or sweat and folding only exert a minor impact on the efficiency of the mask. As a result, masks can be worn for more than the usually recommended 4 h. Also, provided that any potential viral or bacterial contaminations of the mask can be strongly reduced after a given storage time, and that the masks are not visibly soiled or damaged, the strategy to store and reuse surgical masks as proposed by several research groups seems feasible, even if the masks have been handled without particular care, in contact with sweat and saliva, and folded several times.

In parallel, we evaluated the washability of these masks through the same protocol. The effect of washing the mask can be interpreted in light of the European standard EN 14683 and BFE, as reported recently, with the conclusion that the mask still comply after 10 cycles of washing^11^.On the other hand, our study confirmed the risk of reduced efficiency for particle sizes below a few µm^19^. Considering the extended use of the surgical masks and the potential role of fine aerosols in the COVID-19 spread, we suggest that washing the masks should be avoided, even though their mechanical integrity is rather well preserved, as their filtration of fine aerosols is greatly reduced due to disappearance of all electrostatic interactions.

## Materials and methods

### Materials

Type IIR, non-woven polypropylene (PP) premium face masks with elastic earloops from Care & Serve^®^ (WIROS Gmbh, Germany)^20^ were used. They are composed of three layers made of synthetic PP fibres: outer, filter/middle and inner (face contact) layer.

The outer layer is made of hydrophobic non-woven spun-bonded PP which prevents the penetration of droplets through the mask, avoids the inhalation of droplets by the user, and provides splash resistance. The filter, inner, layer is made of a densely packed fine fiber layer of meltblown non-woven PP providing the main filtration and barrier functions, resulting in a bacterial filtration efficiency of 98% for a mean particle size of 3 µm^21^. Furthermore, this layer is electrostatically charged to block very fine particles and viruses. Finally, the inner layer is made of hydrophilic non-woven spun-bonded PP to block the droplets ejected by the user during exhaling, speaking, etc.…, and avoid their penetration through the mask. The spun-bond inner and outer layers also provide the mechanical integrity of the mask. The microstructure of the different layers is presented in Fig. 1a highlighting their difference in pore size and structure.

### Methods

#### Protocols to simulate wear during regular use

##### Folding

Typical use of masks consists of periods of wear often followed by folding and storing the mask in pockets, pouches or elsewhere even if not recommended by the producer. Each cycle of unfolding, use and refolding may cause degradation in mechanical and filtration properties. An experimental protocol was conceived to mimic this cycle: both ends of a mask were taped onto compression platens connected to a Universal Testing Machine (Walter + Bai AG Series LFL-125kN). A transparent plastic cylinder enclosed the mask to ensure it was folded in a random fashion. The folding cycle started with a loosely stretched mask fixed on platens with 165 mm distance between them and consisted of compression at a speed of 10 mm s^−1^ to a final distance of 5 mm. To simulate different extents of use, we varied the number of folding cycles from 10, 20, 50–100 cycles.

##### Ageing

During use, the mask is in contact with body fluids, such as saliva and sweat. To simulate fluid interaction induced mask ageing, a protocol was conceived with artificial sweat or saliva. Artificial sweat was prepared according to the formulation by Ferell et al.^22^, and artificial saliva following Klimek et al.^23^. The masks were aged by immersing in one of the solutions for a pre-determined amount of time that ranged from 1 h to 1 week. Subsequently, the masks were dried at ambient conditions for one day. A set of masks was also subjected to a folding treatment after ageing and drying. The compounds and their providers are listed in Supplementary Tables S1 and S2.

In order to remove all the electrostatic charges and to obtain only a mechanical filtration, some masks were discharged according to EN 779^24^ by isopropanol (IPA) liquid discharging.

##### Combined in-situ treatment

Folding and ageing treatments were performed on separate sets of masks. However, in real life the folding and fluid interaction take place successively. To simulate this, we combined the folding and ageing treatments as follows: the mask underwent several compression/decompression cycles; before each compression step, both sides of the mask were sprayed with either the artificial saliva or the artificial sweat. Once the process was performed the masks were dried under a fume hood.

##### Washing

The washing treatment was performed with detergent powder (10 g/L ECE non-phosphate reference detergent) according to the ISO 6330:2012 but with a household washing machine (Adora L from V-Zug AG) instead of a standardized testing machine. It consisted of 10 washing cycles at 60 °C, for 45 min. In total, 6 masks were washed: three of them were used for the mechanical and air permeability tests; the rest was tested for their filtration efficiency. Table 1 gives an overview of performed treatments and the number of masks treated.

#### Characterization methods

##### Microscopy

A Keyence VHX-7000 microscope equipped with a 100 × lens was used to observe the microstructure of mask layers. Treated and untreated masks were analyzed to assess the effects of the different treatments on the microstructure of the spunbond and meltblown PP layers.

A scanning electron microscope GeminiSEM from Zeiss was used in High Vacuum mode to observe the structure of a reference and a washed mask. Prior to observation, the samples of individual layers were coated with gold to a thickness of 50 nm.

##### Permeability test/breathability test

Breathability was assessed by air permeability measurement using a Textest FX 3320 MOBILAIR test bench. Each specimen was tested at 5 different locations with an area of 5 cm^2^ as shown in Fig. 1b.

Measurement sites were chosen at an adequate distance from the edges of the mask avoiding overlap between the test locations; measurements took place after unfolding the plies. Each site had its air permeability measured at three different pressure levels: 50, 98 and 200 Pa, to fit the permeability as a linear function of the input pressure. From the obtained fit, it was possible to derive the breathability, characterized by the pressure drop, ΔP, according to EN14683, i.e., at a flow rate of 8 L min^−1^ on a surface of 4.9 cm^2^ which is equivalent to an air permeability of the material of 272 L m^−2^ s^−1^. According to this standard, the pressure drop should be below 60 Pa cm^−2^ for Type IIR masks^25^.

Three masks for extreme cases of folding (100 cycles) and ageing (1 week in saliva or sweat) were tested as compared to reference masks.

##### Mechanical characterization

Each layer of reference and treated masks were characterized in uniaxial tensile mode with a Zwick uniaxial testing machine equipped with a 100 N load cell. The samples were extracted by direct die-cutting of the layers in the horizontal direction from individual layers of the studied masks in a dog bone shape with a length of 75 mm and a width of approximately 4 mm as shown in Fig. 1c. Prior to the tests, the exact width and the thickness of the samples were measured using respectively an optical microscope Keyence VHX-7000 and a micrometer screw gauge.

The specimens were tested in tensile mode at a displacement speed of 50 mm min^−1^ until specimen failure during which both the displacement and the force were recorded. From the data acquired, the engineering stress–strain curves of the different specimens were derived. The acquired data were then used to extract the mechanical properties of the three layers of the different masks, such as the tangent Young's modulus taken at 0.2% of strain, the ultimate tensile strength as well as the deformation that the layers undergo before failure.

As for the permeability tests, the three extreme configurations were tested to assess their impact on the mechanical properties of the mask layers. For each configuration and the reference mask, 9 samples from 3 different masks (3 per mask) were cut out.

##### Surface potential measurement

The surface potential of the masks was characterized using an electrostatic voltmeter (Monroe 244A) connected to a computer, as described by He et al.^26^. To perform the measurements, the masks were placed on a grounded metal platform and the probe of the electrostatic voltmeter was set at 5 mm above the specimen. Once all was set, the electrostatic potential of the mask was measured at 5 different positions on the mask to evaluate the mean electrostatic potential of the entire surface of the mask. Each measurement was performed on a circular area with a diameter of 10.5 mm. The side on which the measurement was conducted was shown to affect the results. This characteristic is attributed to the different structure and treatments of the inner and outer layers (hydrophilic vs. hydrophobic). The electrostatic charge of the mask was thus systematically measured from the outer layer, for comparison purposes. The five measurements were performed approximately at the same position as the permeability tests (Fig. 1d).

##### Filtration efficiency (FE)

Circular specimens with a diameter of 60 mm were sampled from masks and hermetically sealed in a sample holder to obtain an effective test surface of 1.66 × 10^3^ mm^2^. An aerosol was drawn with an aerosols generator (AGK2000) from a solution at 0.02 g/mL of fructose in demineralized water. Fructose particles at a concentration of 35 mg m^−3^ in dried air were neutralized in a corona discharging unit (CD2000) and driven to the sample. A constant airflow of 8 L min^−1^ was exerted through the mask specimen (from outside to inside); particle penetration was quantified using the particle analyzer Cambusion DMS500 (see Fig. 1e). This “Fast Particulate Spectrometer” uses unipolar corona charging and parallel detection of particles of varying electrical mobility to offer real-time measurement of the particle size spectrum between 5 and 2500 nm.

The FE is determined by comparing a steady flow of particles after a constant concentration is reached during 2 min with the mask sample, then measuring the raw gas concentration during another minute without the mask sample. FE is expressed as a percentage and is reported in the particle range from 100 to 1200 nm, averaged over triplicates. Therefore, FE here was not measured according to the EN 14683 which requires other particles of 0.6–7 µm in size.

## Supplementary Information

Supplementary Information.

## References

[1] Shereen MA, Khan S, Kazmi A, Bashir N, Siddique R (2020). COVID-19 infection: Emergence, transmission, and characteristics of human coronaviruses. J. Adv. Res..

[2] Huang C (2020). Clinical features of patients infected with 2019 novel coronavirus in Wuhan, China. The Lancet.

[3] Lotfi M, Hamblin MR, Rezaei N (2020). COVID-19: Transmission, prevention, and potential therapeutic opportunities. Clin. Chim. Acta.

[4] Wu, H., Huang, J., Zhang, C. J. P., He, Z. & Ming, W.-K. Facemask shortage and the novel coronavirus disease (COVID-19) outbreak: Reflections on public health measures. Clin. Med. **21**, 100329 (2020). 10.1016/j.eclinm.2020.100329 PMC7129293 32292898

[5] Davies A (2013). Testing the efficacy of homemade masks: Would they protect in an influenza pandemic?. Disaster Med. Public Health Prep..

[6] Clapp PW (2021). Evaluation of cloth masks and modified procedure masks as personal protective equipment for the public during the COVID-19 pandemic. JAMA Intern. Med..

[7] Chughtai AA (2019). Contamination by respiratory viruses on outer surface of medical masks used by hospital healthcare workers. BMC Infect. Dis..

[8] Tcharkhtchi A (2021). An overview of filtration efficiency through the masks: Mechanisms of the aerosols penetration. Bioactive Mater..

[9] Konda A (2020). Aerosol filtration efficiency of common fabrics used in respiratory cloth masks. ACS Nano.

[10] Hossain, E. *et al.* Recharging and rejuvenation of decontaminated N95 masks. *Phys. Fluids* **32**, 093304 (2020). 10.1063/5.0023940 PMC7513826 32982134

[11] Alcaraz, J.-P. *et al.* Reuse of medical face masks in domestic and community settings without sacrificing safety: Ecological and economical lessons from the Covid-19 pandemic. *Chemosphere* **288**, 132364 (2022). 10.1016/j.chemosphere.2021.132364 PMC8491628 34600007

[12] Ekabutr P (2019). Development of antituberculosis melt-blown polypropylene filters coated with mangosteen extracts for medical face mask applications. Polym. Bull..

[13] Drewnick F (2021). Aerosol filtration efficiency of household materials for homemade face masks: Influence of material properties, particle size, particle electrical charge, face velocity, and leaks. Aerosol Sci. Technol..

[14] Manea, L. R., Bertea, A. P. & Bertea, A. Progress in electrospun nanofibres for air filtration. *IOP Conf. Ser.: Mater. Sci. Eng.* **877**, 012043 (2020).

[15] Biermann, A. H., Lum, B. Y. & Bergman, W. Evaluation of permanently charged electrofibrous filters. (1982).

[16] Kim J, Hinestroza JP, Jasper W, Barker RL (2009). Effect of solvent exposure on the filtration performance of electrostatically charged polypropylene filter media. Text. Res. J..

[17] Bouchet, A. *et al.* What is the environmental impact of different strategies for the use of medical and community masks? Preprints (2021).

[18] Schmutz M (2020). Cotton and surgical masks—What ecological factors are relevant for their sustainability?. Sustainability.

[19] Chua MH (2020). Face masks in the new COVID-19 normal: Materials, testing, and perspectives. Research.

[20] BSI British Standards. *Medical face masks—Requirements and test methods:* https://linkresolver.bsigroup.com/junction/resolve/000000000030401487?restype=standard. 10.3403/30359568.

[21] F23 Committee. *Specification for performance of materials used in medical face masks*. http://www.astm.org/cgi-bin/resolver.cgi?F2100-20. 10.1520/F2100-20.

[22] Ferrell, J. K., Branscome, M. R. & Rousseau, R. W. *Perspiration poisoning of protective clothing materials. Part I. Mathematical model for a complex adsorption bed*. **55** (1974).

[23] Klimek J, Hellwig E, Ahrens G (1982). Fluoride taken up by plaque, by the underlying enamel and by clean enamel from three fluoride compounds in vitro. Caries Res..

[24] EN 779. *Particulate air filters for general ventilation—Determination of the filtration performance* (2012).

[25] CEN. *Community face coverings—Guide to minimum requirements, methods of testing and use* (2020).

[26] He, W. *et al.* Filtration performance and charge degradation during particle loading and reusability of charged PTFE needle felt filters. *Sep. Purific. Technol.* **233**, 116003 (2020).

