# Supplementary material for: On the durability of surgical masks after simulated handling and wear
Source: Sci Rep. 2022 Mar 23;12:4938. doi: 10.1038/s41598-022-09068-1 (PMC8943131; doi:10.1038/s41598-022-09068-1)
Supplement: Supplementary file 1 — Supplementary Information. [file 41598_2022_9068_MOESM1_ESM.docx]

# On the durability of surgical masks after simulated handling and wear

Vincent Varanges^1^; Baris Caglar^1,2^; Yann Lebaupin^1^; Till Batt^3^; Weidong He^4, 5^; Jing Wang^4,5^; René M Rossi^3^; Gilles Richner^6^; Jean-Romain Delaloye^7^; Véronique Michaud^1^*

^1^Laboratory for Processing of Advanced Composites (LPAC), Institute of Materials (IMX), Ecole Polytechnique Fédérale de Lausanne (EPFL), Station 12, Lausanne CH-1015, Switzerland

^2^Aerospace Manufacturing Technologies, Faculty of Aerospace Engineering, Delft University of Technology, Kluyverweg 1, Delft 2629HS, the Netherlands

^3^ Biomimetic Membranes and Textiles Laboratory, Swiss Federal Laboratories for Materials Science and Technology (Empa), CH-9014 St. Gallen, Switzerland

^4^Laboratory of advanced analytical technologies, Swiss Federal Laboratories for Materials Science and Technology (Empa), Dübendorf, CH-8600, Switzerland

^5^Institute of Environmental Engineering, ETH Zürich, Zürich, CH-8093

^6^Federal Office for Civil Protection FOCP, Spiez Laboratory, Spiez, Switzerland

^7^Clinic of Orthopaedics and Traumatology, Department of Surgery, Kantonsspital, Winterthur, Switzerland

*Corresponding author. Email-address: veronique.michaud@epfl.ch

Supplementary figures and tables.


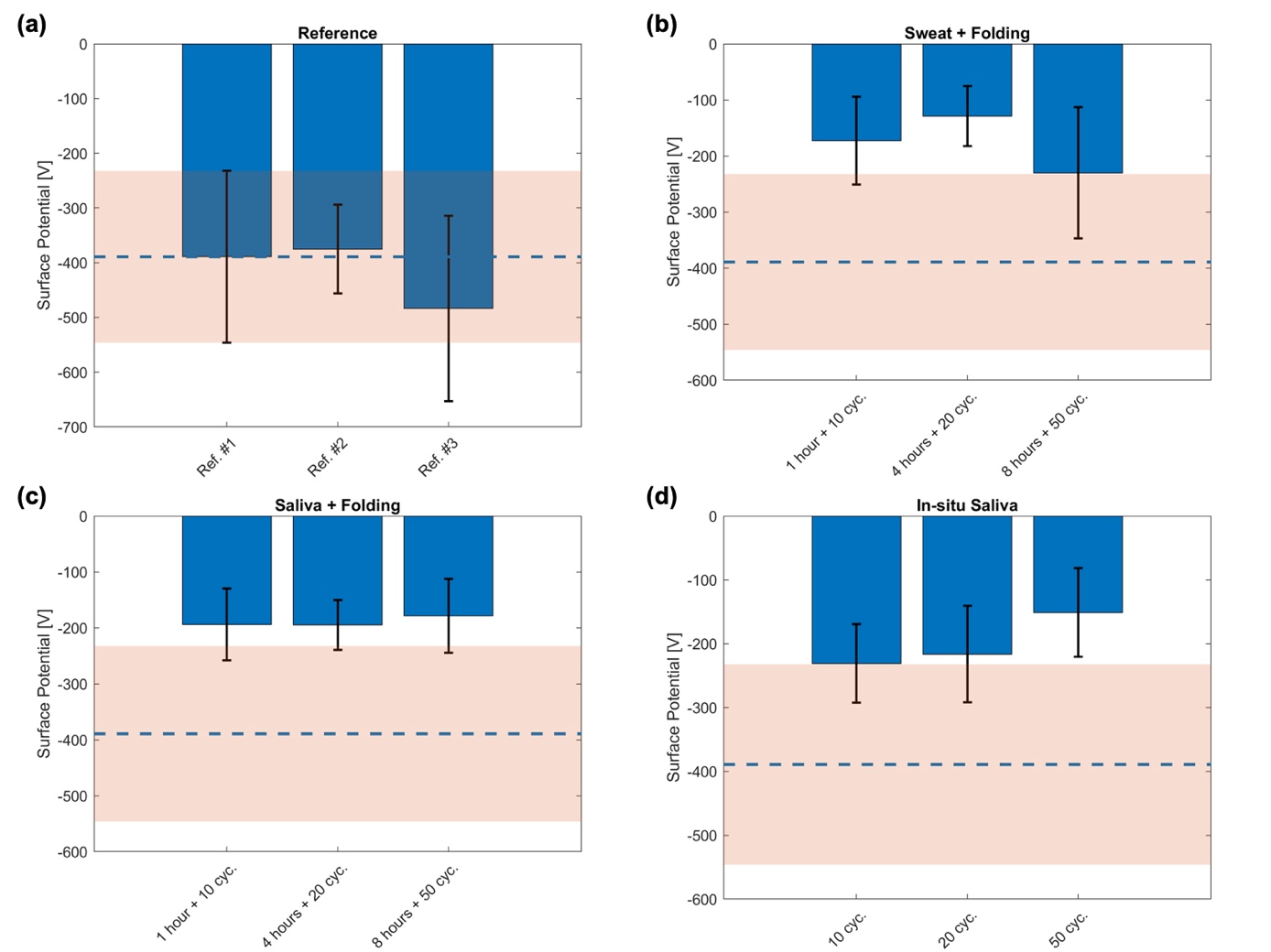


**Supplementary Figure S1: Continuation of Figure 3 for the results from the characterization of the surgical mask’s surface potential (electrostatic potential)**: For each figure the reference data are used as a comparison and are represented by the dashed blue line with its standard deviation in light orange shaded area for the surface potential histograms. a) for the reference masks; b) the combination of ageing in sweat and folding; c) the combination of ageing in saliva and folding; d) the in-situ treatment with saliva.


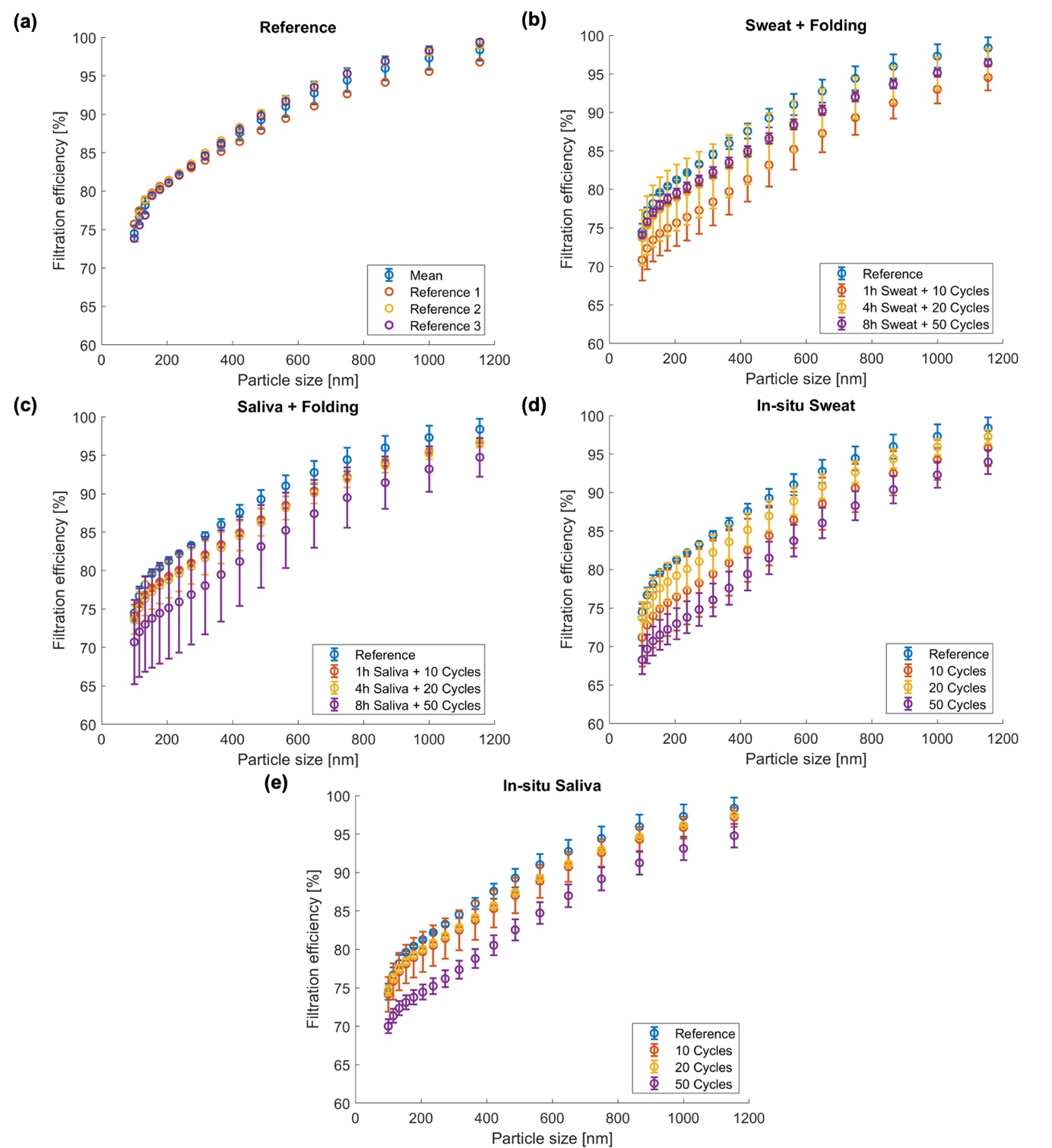


**Supplementary Figure S2: Continuation of Figure 4 for the results from the characterization of the surgical mask’s filtration efficiency after a set of different treatments in order to assess their degree of degradation and so their durability after a given time of use**: For each figure the reference data are used as a comparison and are represented by the series of blue points: a) for the reference masks; b) the combination of ageing in sweat and folding; c) the combination of ageing in saliva and folding; d) the in-situ treatment with sweat and e) the in-situ treatment with saliva.

Supplementary Table S1: List of the compound used for the preparation of the artificial sweat with their provider

| Compound | Name | Provider |
| --- | --- | --- |
| NaCl | Sodium Chloride | Fluka |
| K_2_SO_4_ | Potassium Sulfate | Fischer Chemical |
| Na_2_SO_4_ | Sodium Sulfate | Sigma-Aldrich |
| MgSO_4_ | Magnesium Sulfate | Reactolab |
| CaCl_2_ | Calcium Chloride | Sigma-Aldrich |
| CH_4_N_2_O | Urea | Acros Organics |
| C_6_H_12_O_6_ | Glucose | Sigma-Aldrich |
| C_3_H_6_O_3_ | Lactic Acid | Acros Organics |
| C_3_H_4_O_3_ | Pyruvic Acid | Acros Organics |
| NH₄OH | Ammonium Hydroxide | Acros Organics |
| H_2_O | Distilled water | Laboratory Millipore filter |

Supplementary Table S2: List of the compound used for the preparation of the artificial saliva with their provider

| Compound | Name | Provider |
| --- | --- | --- |
| C_6_H_8_O_6_ | L-Ascorbic acid | Sigma-Aldrich |
| C_6_H_12_O_6_ | Glucose | Sigma-Aldrich |
| NaCl | Sodium Chloride | Fluka |
| CaCl_2_ | Calcium Chloride | Sigma-Aldrich |
| KCl | Potassium chloride | Sigma-Aldrich |
| KSCN | Potassium thiocyanate | Sigma-Aldrich |
| KH_2_PO_4_ | Potassium phosphate monobasic | Sigma-Aldrich |
| CH_4_N_2_O | Urea | Acros Organics |
| Na_2_HPO_4_ | di-Sodium hydrogen phosphate | Sigma-Aldrich |
| Mucin | Mucin from porcine stomach Type II | Sigma-Aldrich |
| H_2_O | Distilled water | Laboratory Millipore filter |
